# Unusual Axillary Recurrence Observed in Breast Cancer With Bloody Nipple Discharge: A Case Report

**DOI:** 10.7759/cureus.82643

**Published:** 2025-04-20

**Authors:** Haruka Jono, Shoji Oura, Kiyoshi Yoshikawa

**Affiliations:** 1 Department of Surgery, Kishiwada Tokushukai Hospital, Kishiwada, JPN; 2 Department of Surgery, Suita Tokushukai Hospital, Suita, JPN

**Keywords:** axillary recurrence, bloody nipple discharge, breast cancer, cancer cell dissemination, indistinct margins

## Abstract

After breast cancer surgery, a major part of axillary recurrences occur in lymph nodes. We report a case of breast cancer in which bloody nipple discharge spillage onto the surgical field was possibly associated with axillary recurrence. A 47-year-old woman with a history of mastectomy and sentinel node biopsy noticed an axillary mass. Her luminal breast cancer had been detected by bloody nipple discharge and pathologically diagnosed as a predominantly intraductal carcinoma seven years before. The axillary lesion was depicted as an oval mass both with indistinct margins and predominant internal high echoes on ultrasound. Magnetic resonance imaging of the mass showed slightly low signals on T1-weighted images and faint high signals on fat-suppressed T2-weighted images. Positron emission tomography showed no avid fluorodeoxyglucose uptake in the axillary mass. Due to the proven cytological malignancy of the axillary mass, the patient underwent salvage axillary dissection under the tentative diagnosis of axillary node recurrence. Post-operative pathological study, however, showed that the mass had atypical cells growing in cribriform and tubular fashions in the fibro-fatty tissue and did not have any lymph node structures, noninvasive cancer components, lympho-vascular involvement, normal mammary gland components, and metastatic foci in the dissected lymph nodes. These results suggested an association between bloody nipple discharge spillage onto the surgical field and axillary recurrence. The patient has been well on adjuvant leuprorelin and letrozole therapy for six months. Breast surgeons should note that bloody nipple discharge spillage during the operation may be a risk factor for local recurrence.

## Introduction

Axillary lymph node dissection can provide breast oncologists with information about lymph node metastasis, which is useful for selecting more appropriate adjuvant therapy. On the other hand, routine axillary lymph node dissection had markedly impaired the quality of life of many breast cancer patients for more than a century [1]. However, widespread use of sentinel lymph node biopsy (SNB) [2] has dramatically reduced the number of patients suffering from axillary lymph node dissection-related unpleasant complications such as lymphedema and paresthesia [3]. Conversely, SNB has been causing much more axillary recurrence than the standard axillary dissection did [4]. All breast surgeons, therefore, must pay close attention to the early detection of axillary recurrence after SNB, like in-breast recurrence after breast-conserving therapy [5].

Lymph node recurrence accounts for the major part of axillary recurrence after breast cancer surgery. Naturally, breast cancer patients can have non-lymphatic axillary recurrence [6,7]. Possible non-lymphatic axillary recurrence includes breast cancer arising from some kind of mammary gland present in the axilla and cancer cell dissemination to the axillary surgical field [8]. Delayed diagnosis of axillary recurrence naturally has the potential to aggravate the post-relapse outcome of the patients. Breast specialists, however, rarely encounter non-lymphatic axillary recurrences and generally do not even know whether the diagnosis of them is easy or not.

Bloody nipple discharge is a relatively common symptom of breast cancer. The presence of bloody nipple discharge suggests the presence of intraductal cancer cells near the nipple, and is a symptom that may have some impact on surgical options for breast cancer. However, no studies have reported the correlation between bloody nipple discharge and local recurrence to date.

We herein report a case of breast cancer detected by bloody nipple discharge, which post-operatively developed non-lymphatic axillary recurrence with extremely rare image findings.

## Case presentation

**Figure 1:**
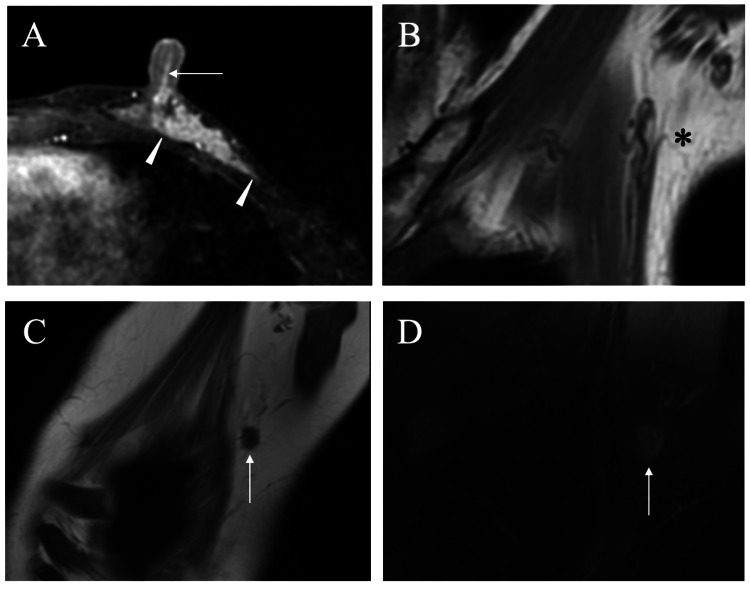
Magnetic resonance imaging (MRI) findings A. Subtraction MRI at the primary operation showed extremely wide enhancement, i.e., more than 40 mm (arrowheads), in the left mammary gland including intra-nipple ducts (arrow) by presumed ductal spread. B. MRI at the primary operation did not show any mammary glands in the axilla (asterisk). C. MRI of the axillary mass showed low signals on T1-weighted images. D. T2-weighted MRI images of the axillary mass only showed faint high signals.

A 47-year-old woman presented to a hospital due to bloody nipple discharge at the age of 40. No malignant findings were detected on mammography. However, cytological study of the bloody nipple discharge revealed malignant cells. Magnetic resonance imaging (MRI) showed extensive enhancement in the mammary gland, presumably due to wide ductal spread and no image detectable mammary glands in the axilla (Figure 1A, 1B).

**Figure 2:**
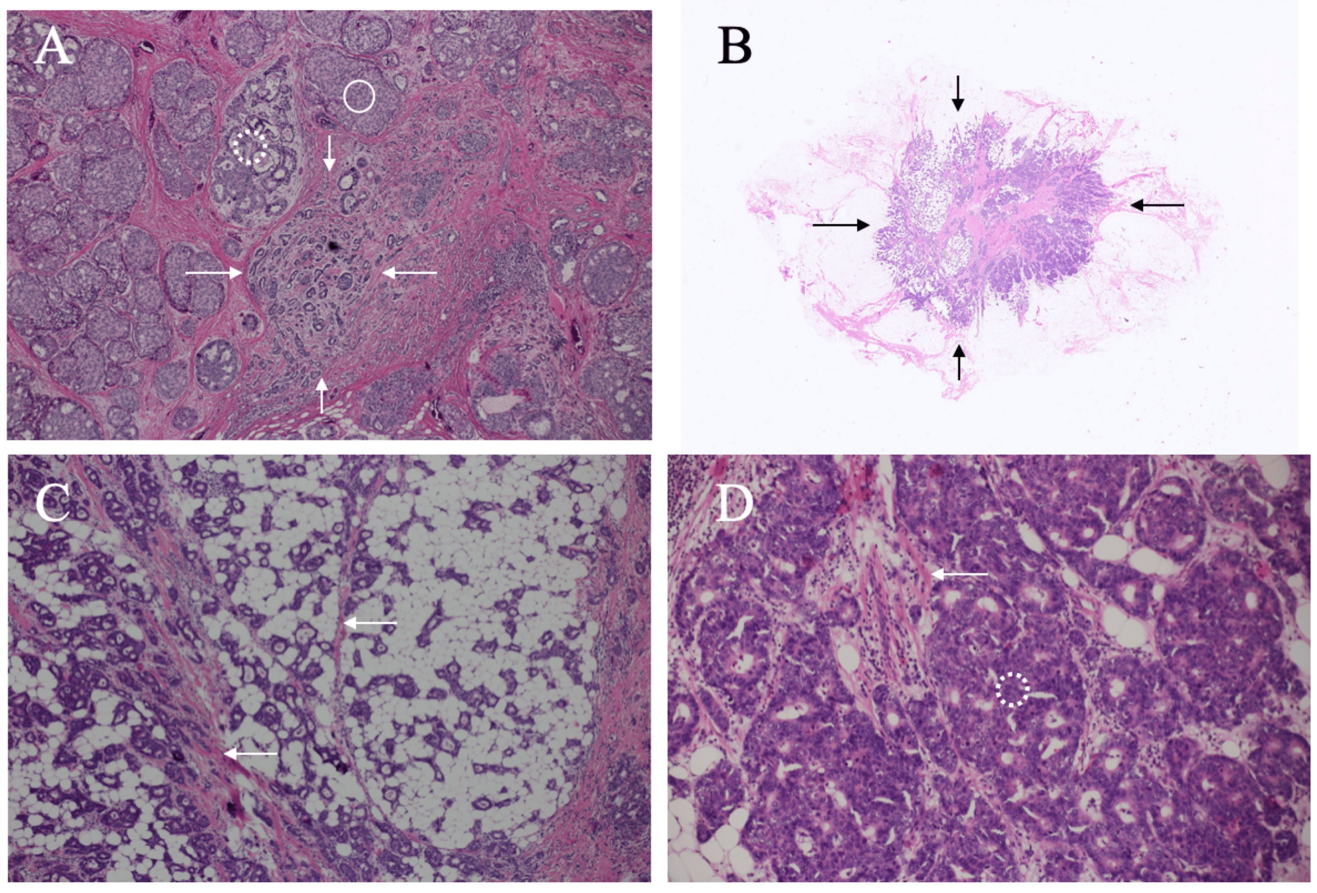
Pathological findings A. The primary breast cancer showed non-invasive cancer cells growing in solid (open circle) and cribriform (dotted open circle) fashions and very small invasive cancer clusters, 6 mm in size (arrows) (H.E. ×100). B. Low magnified view showed an oval mass (arrows), approximately 1 cm in size, with atypical cells in fibro-fatty tissue. No lymph node and mammary gland structures were observed (H.E. ×20). C. Magnified view (H.E. ×100) showed atypical cells growing sparsely in the fibro-fatty tissue. Cancer cell clusters did not have fibrous components around or in them, but rather were present around the fibrous components (arrows). D. Magnified view (H.E. ×200) showed atypical cells growing in a cribriform fashion (dotted open circle) with a small amount of fibrous components (arrow) in the cell-rich areas.

The patient, therefore, underwent total mastectomy and sentinel node biopsy using methylene blue and Tin colloid. Unfortunately, details of bloody nipple discharge management during the operation was uncertain due to the lack of information about the nipple discharge in the operation record. Post-operative pathological study showed that predominantly intraductal carcinoma cells (nuclear grade 1), i.e., a maximal invasive cancer size of 6 mm (pT1b), grew in cribriform and solid fashions (Figure 2A) which spread widely (total length > 40 mm) in the mammary gland and had neither lymphatic metastasis (pN0) nor positive margins.

**Figure 3:**
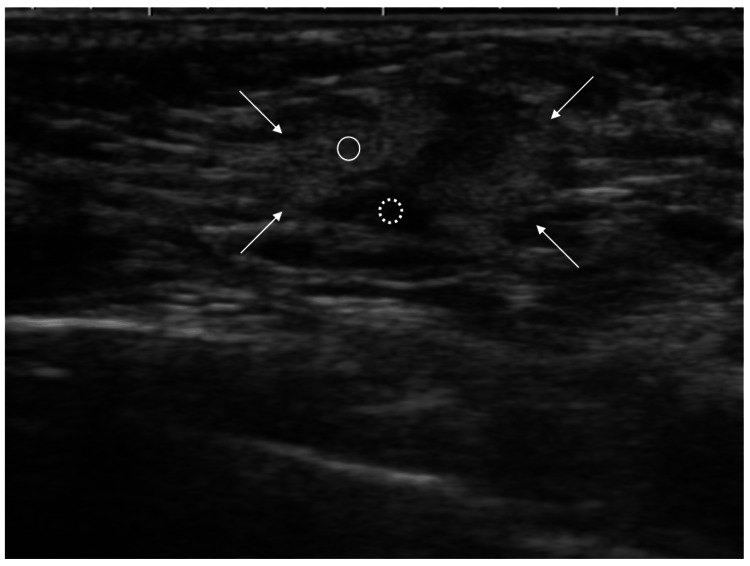
Ultrasound findings Ultrasound showed an oval mass both with indistinct margins (arrows) and mixed high (open circle) and low (dotted open circle) internal echoes in the axilla. When compared with the low-magnified pathological image (Figure 2B), the low echo areas corresponded to fibrous components, while the high echo areas reflected that cancer cells were scattered in fat with low acoustic impedance, generating a large amount of backscattering.

Immunostaining showed that her breast cancer had estrogen receptor (ER) and progesterone receptor (PgR) positivities (both Allred score 8), human epidermal growth factor receptor type 2 (HER2) negativity, and a low Ki-67 labelling index of 5%. In seven years on adjuvant tamoxifen therapy, the patient noticed a small mass in her left axilla. Ultrasound showed an oval mass, 12 mm in size, both with indistinct margins and predominant internal high echoes (Figure 3).

**Figure 4:**
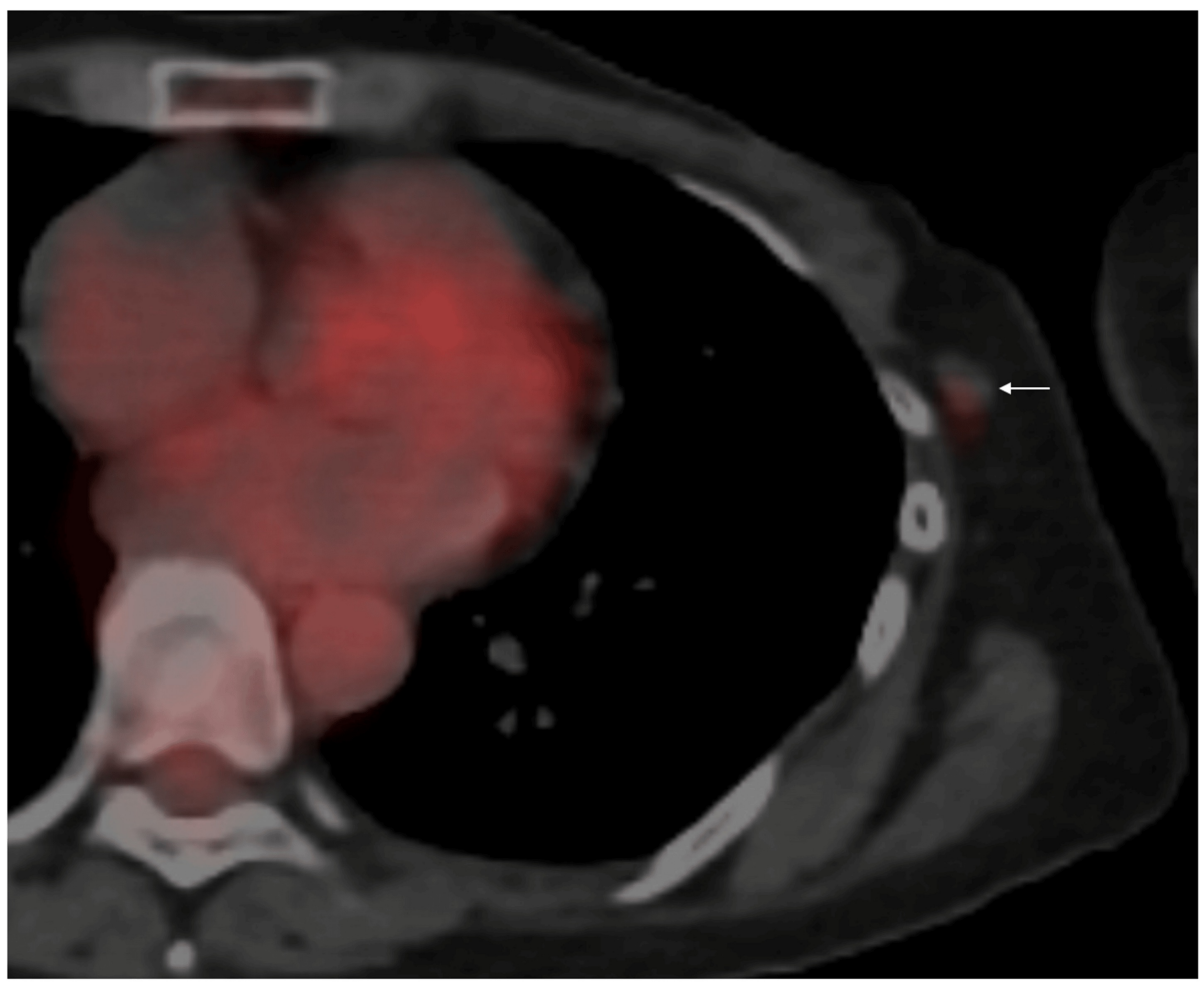
Positron emission tomography (PET)/computed tomography (CT) findings PET/CT showed no avid radio tracer uptake (arrow) in the axillary mass, strongly suggesting that malignant cells, even if present, were very sparsely present in the target lesion.

MRI of the axillary mass showed slightly low signals on T1-weighted images and faint high signals on fat-suppressed T2-weighted images (Figure 1C, 1D). Positron emission tomography showed no avid fluorodeoxyglucose uptake in the axillary mass (Figure 4).

Due to the proven cytological malignancy of the axillary mass, the patient underwent salvage axillary dissection under the tentative diagnosis of axillary lymph node recurrence. Post-operative pathological study, however, showed that the tumor had atypical cells growing in cribriform and tubular fashions in the fibro-fatty tissue and did not have any lymph node structures, noninvasive cancer components, lymph-vascular involvement, normal mammary gland components, and metastatic foci in the dissected lymph nodes (Figure 2B-2D). Immunustaining of the tumor showed ER (Allred score 8) and PgR (Allred score 6) positivities, HER2 negativity, and a high Ki-67 labelling index of 55%. Due to the patient’s preference, the patient has been receiving adjuvant leuprorelin and letrozole therapy without any recurrence for six months.

## Discussion

Internal echoes of a mass are determined by the presence and degree of ultrasound wave backscattering. Ultrasound waves hardly backscatter in a mass consisting exclusively of tumor cells with similar acoustic impedance and therefore make internal echoes of the mass very low, often observed in malignant lymphomas and medullary breast carcinomas. Conversely, due to different acoustic impedance, ultrasound waves backscatter in a mass either with papillary/tubular structures or interminglement of different substances/cells, leading to high internal echo formation [9-11]. This case had both cribriform (a subtype of papillary structures)/tubular structures and interminglement of fat cells within the mass. The lack of normal mammary gland components in the recurrent nodule suggested that this recurrence did not develop in some kind of axillary mammary glands [6,7].

The recurrent focus in this case had unclear mass margins and contained a relatively large amount of fibrous components. Dvorak et al. [12] define cancer as the tissue of "wounds that do not heal”, which endlessly produce cancer-associated fibroblasts to repair the wounds. In other words, fibrosis around or in cancer cell clusters occurs in order to repair tissue damage caused by cancer cell proliferation. However, judged by the pathological findings, the fibrosis observed in this case seemed to be, at least partially, formed for wound healing after the primary operation followed by cancer cell proliferation around it, rather than repairing the tissue surrounding the cancer cells.

No studies have reported that spillage of bloody nipple discharge, containing cancer cells, onto the surgical field is a cause of local recurrence. It, however, is well known that needle tract cancer cell seeding can be a risk factor for local recurrence in various cancers including breast cancer [13,14]. In addition, the mechanism of Kruckenberg ovarian tumor formation [15] seems very similar to the nipple discharge-related local recurrence.

The cancer milieu in the axilla, which differed from that of the primary tumor, might have been associated with the higher Ki67 labeling index [16] in the recurrent focus. Possible mechanisms for this biological change include the heterogeneity and phenotypic alteration of cancer cells. However, we naturally cannot determine which mechanism is responsible for the change in this case. It, therefore, is imperative that the mechanism of the marked increase of Ki-67 labelling index in the locally recurrent lesion be investigated in the future.

Incidental palpation of the axillary mass by the patient herself fortunately led to the diagnosis of breast cancer recurrence. Without the patient's complaint, it would have been very difficult for us to identify the axillary recurrence even using any diagnostic images. Breast surgeons should be aware that bloody nipple discharge, when spilled onto the operative field, could be a risk factor for local recurrence. In addition, diagnostic physicians should note that cancer cell growth in a sparse fashion can result in non-lymphatic axillary recurrence with extremely rare image findings. Therefore, on performing surgery for breast cancer with bloody nipple discharge, it is recommended that the surgical field be irrigated with saline or distilled water [17] before skin closure.

## Conclusions

It is commonly recognized by many oncologists that tumor cell leakage into the surgical field is a risk factor for local recurrence in any cancer. We, therefore, cannot rule out the possibility that bloody nipple discharge spillage onto the surgical field could affect the local recurrence in this case. Breast surgeons should keep this rare recurrence mechanism in mind when performing surgery and note the possible axillary recurrence that is difficult to diagnose with any imaging modalities on follow-ups. In addition, we recommend that the surgical field of breast cancers with cancer cell-containing bloody nipple discharge be irrigated either with saline or, if possible, distilled water just before the end of surgery to avoid unpleasant axillary recurrence.
